# Streamlining R&D permission for substantial amendments to oncology clinical trials at an NHS site: a case study

**DOI:** 10.1186/1745-6215-16-S2-P190

**Published:** 2015-11-16

**Authors:** David I'Anson, Amy Smith, Natasha Aslam, Kylie Gyertson, Rajinder Sidhu, Smaragda Agathou, Ashparan Gill, Jeremy Whelan

**Affiliations:** 1 University College London Hospitals NHS Foundation Trust, London, UK

## Background

Trust approvals for substantial amendments at NHS sites are a notoriously lengthy process, impacting on the ability to adapt to changes quickly, at the cost of potential recruitment opportunities.

## Aims

To reduce ‘time to approval’ whilst maintaining accurate and safe reviews.

## Methods

Observation and mapping of Trust wide processes to identify shortfalls and barriers to timely approval. Evident that capacity within service support departments, R&D and local research teams was limited when dealing with amendments from all areas of research. Cases were identified in which not all parties were aware of amendments. It was decided to divide the SOP guided facilitation of service support department sign off (pharmacy, radiology, costing, contracts etc.) between the relevant research units, building capacity for timely reviews and approvals.

## Results

Prior to re-design: Average time from receipt of amendment to approval = 40.71 {2-130} days (n=113); after 7 week pilot: Average time from receipt of amendment to approval = 17.78 {1-40} days (n=26). Time to approval decreased by 55.49%. Furthermore, increased safety through a two tier review of amendments (department and R&D) ensured clinical teams were aware of changes promptly.

## Conclusions

Time to approval has been reduced. Studies can continue with minimal disruption and maximise recruitment opportunities, without compromising the safety and accuracy of reviews. Development of a solid, robust process, which has promoted effective communication and cooperation, has led to this success. This will be further enhanced by the upcoming HRA changes aimed at streamlining governance on a national scale.

